# Fundamental Differences in Inactivation Mechanisms of *Escherichia coli* O157:H7 Between Chlorine Dioxide and Sodium Hypochlorite

**DOI:** 10.3389/fmicb.2022.923964

**Published:** 2022-06-17

**Authors:** David F. Bridges, Alison Lacombe, Vivian C. H. Wu

**Affiliations:** Produce Safety and Microbiology Research Unit, Western Regional Research Center, Agricultural Research Service, United States Department of Agriculture, Albany, CA, United States

**Keywords:** chlorine dioxide, sodium hypochlorite, oxidative stress, *Escherichia coli* O157:H7, cellular oxidation

## Abstract

Chlorine dioxide (ClO_2_) and sodium hypochlorite (NaClO) are two chlorinated oxidizing agents that are implemented in water treatment and postharvest processing of fresh produce. While the antibacterial mechanisms of NaClO have been investigated, there are comparatively few studies that have looked at how ClO_2_ kills bacteria. Therefore, the objective of this study was to compare the inactivation pathways of ClO_2_ and NaClO against *Escherichia coli* O157:H7. Treatments consisted of 2.5, 5, and 10 ppm ClO_2_ or 50, 100, and 200 ppm NaClO for 5, 10, and 15 min. Maximum log reductions of *E. coli* O157:H7 were 5.5 and 5.1 after treatment with ClO_2_ or NaClO, respectively. Bacterial inactivation was measured using log reductions, intracellular reactive oxygen species (ROS) using with 2′,7′–dichlorofluorescin diacetate (DCFDA) or aminophenyl fluorescein (APF) probes, relative values of NAD^+^, NADH, NADP^+^, and NADPH cofactors. Additionally, the expression of three key genes involved in ROS stress was measured *via* RT-PCR. Levels of intracellular ROS measured by DCFDA after ClO_2_ treatment were significantly higher than those found after treatment in NaClO. Additionally, NaClO treatment resulted in upregulation of ROS-defense genes, while expression of the same genes was typically at base levels or downregulated after ClO_2_ treatment. As the concentrations of both treatments increased, the NADP^+^:NADPH ratio shifted to the cofactor being predominantly present as NADP^+^. These data indicate that ClO_2_ and NaClO damage *E. coli* O157:H7 *via* measurably different mechanisms and that ClO_2_ does not appear to cause substantial oxidative stress to *E. coli* O157:H7 directly.

## Introduction

Public health experts recognize agricultural and municipal water as a potential vector of enteric pathogens. Chlorinated sanitizers such as sodium hypochlorite (NaClO) and chlorine dioxide (ClO_2_) are commonly used as antimicrobials for wastewater sanitation, surface decontamination, and agricultural water treatment. Industries rely heavily on NaClO as their primary sanitizer because of its relatively low cost. While treatments with NaClO have been demonstrated on numerous occasions to reduce microbial populations, the overuse of NaClO has significant drawbacks, creating a need for suitable alternatives. From an efficacy standpoint, NaClO treatments deteriorate outside a narrow pH range and in environments with high organic loads, creating significant issues for the food industry (Goodburn and Wallace, 2013). Additionally, the formation of carcinogenic byproducts and the potential ability to increase the spread of antibiotic-resistance genes has led to occupational and consumer safety concerns (Deborde and von Gunten, 2008; Liu et al., 2018). One potential alternative to NaClO is ClO_2_ which has recently expanded to be utilized as a sanitizer in the food industry (Ofori et al., 2018). Numerous studies have demonstrated that the ClO_2_ disinfection properties are comparable or superior to that of NaClO, especially in situations of higher organic load (Gómez-López et al., 2009; Wu, 2016; Bridges and Wu, 2018; Praeger et al., 2018; Tadepalli et al., 2018; Bridges et al., 2020). Additionally, ClO_2_ has demonstrated more targeted damage to pathogens and subsequent inactivation than NaClO (Praeger et al., 2018). Despite these observations, the antimicrobial mechanisms of ClO_2_ compared to NaClO are poorly understood.

The mechanisms in which NaClO inactivates microorganisms are well known and have been described in great detail (Rodgers et al., 2004; Fukuzaki, 2006; Rajkovic et al., 2010; Gray et al., 2013). In aqueous solutions, NaClO dissociates into hypochlorous acid (HOCl) and hypochlorite (OCl^–^). Because HOCl is uncharged, it is membrane permeable, while the charged OCl^–^ cannot permeate undamaged cell membranes (Fukuzaki, 2006). This makes the antibacterial properties of NaClO largely attributed to the concentration of HOCl in a solution. Once present, HOCl will rapidly react with proteins with sulfur-containing amino acids, such as cysteine or methionine, forming highly reactive intermediates, leading to protein inactivation or degradation (Gray et al., 2013). Additionally, HOCl can also react with nucleotides and lipids, albeit less preferentially than amino acids, leading to DNA and RNA strand breakage, lipid peroxidation, and other damages that can contribute to cell death (Niki, 2009; Stanley et al., 2010; Gray et al., 2013). Although HOCl can damage bacterial cells through numerous mechanisms, it is likely that lethality occurs *via* damage resulting in the inhibition of ion, metabolite, and protein transport across bacterial membranes (Albrich et al., 1986; McKenna and Davies, 1988; Gray et al., 2013).

Chlorine dioxide is another chlorinated sanitizer that is commonly used in water treatment (Gómez-López et al., 2009; Wu, 2016; Bridges and Wu, 2018). Like HOCl, ClO_2_ has demonstrated powerful antimicrobial properties, and numerous studies and reviews described its antibacterial efficacy (Rodgers et al., 2004; Gómez-López et al., 2009; Wu, 2016; Bridges and Wu, 2018; Praeger et al., 2018; Sun et al., 2019). However, there are few studies that have investigated the molecular inactivation pathways of ClO_2_ against pathogens. The putative mechanism of ClO_2_ inactivation involves a similar pathway to HOCl, reacting with sulfur-containing amino acids and proteins (Berg et al., 1986; Praeger et al., 2018) causing increased membrane permeability and subsequent cell death. However, multiple studies that have visually examined cells after ClO_2_ treatment found no obvious cellular deformities or cell wall damage (Ofori et al., 2017, 2018; Bridges et al., 2020). Exemplifying this, Bridges et al. (2020) demonstrated that treatment of *Escherichia coli* O157:H7 with concentrations of ClO_2_ ≤ 15 ppm did not significantly interfere with the maintenance of membrane polarity, indicating that damages from ClO_2_ were not localized at the membrane level (10). These results suggest that lethal damages caused by ClO_2_ are not superficially localized, and routes of ClO_2_-inactivation could be intracellular.

Despite both HOCl and ClO_2_ being chlorine-based oxidizers, it is plausible that the responses that bacteria employ when responding to each sanitizer are measurably different. By examining how both chlorinated sanitizers affect multiple physiological metrics, fundamental differences in antimicrobial mechanisms might become apparent. Therefore, the objective of this study was to evaluate *E. coli* O157:H7 after exposure to either NaClO or ClO_2_ to determine how ClO_2_ affects bacteria cells differently from NaClO. Specifically, levels of intracellular reactive oxygen species (ROS), expression of select genes, and the state of specific cofactors were measured to highlight important differences in how each sanitizer kills bacterial cells.

## Materials and Methods

### Preparation of Bacteria

*Escherichia coli* O157:H7 (ATCC^®^ 35150) was maintained at −80°C throughout the study. Due to differences between strains, only one strain of *E. coli* was selected for use to reduce variance in collected data. Before experimentation, 10 ml of tryptic soy broth (TSB) was inoculated using a culture maintained in frozen conditions and incubated overnight at 37°C. This first culture was then used to inoculate a second 10 ml tube of TSB and was incubated overnight at 37°C. Following incubation, the culture was streaked onto slants of tryptic soy agar (TSA), incubated overnight at 37°C, and transferred to storage at 4°C to serve as working stocks. The day prior to experimentation, cultures maintained at 4°C were used to inoculate 10 ml tubes of TSB incubated for 16–18 h overnight at 37°C. Following incubation, the cultures were centrifuged for 10 min at 10,000 × *g*, and the resultant pellet was washed twice with peptone water (0.1%) and resuspended in 9 ml of peptone water.

### Preparation of Aqueous Sanitizers

Stock solutions of NaClO and ClO_2_ were prepared as described previously (Tadepalli et al., 2018; Bridges et al., 2019). In short, working solutions of 500, 1,000, and 2,000 ppm NaClO were made by diluting bleach solution (6% NaClO) with sterile deionized (DI) water on the day of experimentation. A stock solution of ClO_2_ was generated using a dry media method provided by ICA TriNova, LLC (Forest Park, GA, United States). The generated ClO_2_ stock solution was diluted with DI water to generate working solutions of 25, 50, and 100 ppm ClO_2_ on the day of experimentation, and ClO_2_ was measured by the DPD (N, N-diethyl-r-phenylenediamine) method using a Hatch DR 900 colorimeter as utilized in previous studies (Wu and Kim, 2007; Tadepalli et al., 2018; Bridges et al., 2019).

### Treatment of *Escherichia coli* O157:H7 With Sodium Hypochlorite or Chlorine Dioxide and Measurement of Viability

Due to the fact that NaClO and ClO_2_ are known to have different oxidative strengths and dissimilar antibacterial efficacies at comparable concentrations, treatment concentrations and times were selected based on commonly utilized conditions in published literature and preliminary trials aimed at achieving similar ranges of *E. coli* O157:H7 reduction (Tadepalli et al., 2018, 2019; Bridges et al., 2019). One milliliter of NaClO solution (500, 1,000, or 2,000 ppm) or ClO_2_ solution (25, 50, or 100 ppm) was added to 9 ml of ∼8 log CFU/ml *E. coli* O157:H7 culture to make final concentrations of 50, 100, or 200 ppm NaClO and 2.5, 5, or 10 ppm ClO_2_. Treatments lasted for 5, 10, or 15 min, and the bacterial cultures were vortexed every 5 min. At the end of the treatment, 1 ml of 1% sodium thiosulfate (Na_2_S_2_O_3_) was added to the culture, making a final solution of 0.09% to inactivate any remaining sanitizer as utilized previously (Bridges et al., 2020). A treatment with sterile DI water was included as a control in every experiment. The cultures were then serially diluted in peptone water and plated on MacConkey’s sorbitol agar supplemented with 0.05 mg/l cefixime and 2.5 mg/l potassium tellurite (CT-SMAC) and overlaid with TSA [Thin Agar Layer (TAL) method] to aid in the recovery of sub-lethally injured bacteria as described previously (Wu, 2008).

### Measurement of Intracellular Reactive Oxygen Species With 2′,7′–Dichlorofluorescin Diacetate or Aminophenyl Fluorescein

Levels of intracellular ROS were measured using the DCFDA and APF probes from Life Technologies (Carlsbad, CA, United States). For the ROS assays, one ml of *E. coli* O157:H7 was incubated in 20 μM DCFDA for 1 h at 37°C in darkness or 10 μM APF at room temperature for 30 min in darkness as described previously (Cossu et al., 2017). After incubation, the bacterial cells were centrifuged for 3 min at 10,000 × *g*, washed twice, and resuspended in 900 μL of peptone water. One hundred microliters of ClO_2_ or NaClO were then added to the bacterial culture to start experiments. After the treatment time, 100 μl of Na_2_S_2_O_3_ was added to inactivate the remaining sanitizer. The treated cells were centrifuged for 3 min at 10,000 × *g* and resuspended in peptone water. Two hundred microliters of bacteria were then transferred to clear-bottomed, black-sided 96-well plates, and fluorescence intensity was measured at excitation/emissions of 495/527 nm or 490/515 nm for DCFDA and APF, respectfully, using a SpectraMax M2 Microplate Reader (Molecular Devices, San Jose, CA, United States).

### Expression of Select Genes After Treatment With Sodium Hypochlorite or Chlorine Dioxide

Bacterial RNA was extracted after 15 min treatments and neutralization with Na_2_S_2_O_3_ using a *Quick*-RNA Fungal/Bacterial Kit (Zymo Research, Irvine, CA, United States) with an additional DNase treatment to remove genomic DNA. Expression of superoxide dismutase (*sodA*), hydrogen peroxide-inducible genes activator (*oxyR*), and redox-sensitive transcriptional activator (*soxR*) were selected as key genes to represent oxidative stress response due to their well-known involvement in ROS defense processes (24, 34, 35). Additionally, expression of universal stress protein A (*uspA*), RNA polymerase σ factor (*rpoS*), and outer membrane porin C (*ompC*) were selected to represent the response to general stress, and 16S rRNA served as the controller gene. Primer sequences were selected from previous studies (Mei et al., 2015; Yang et al., 2018) and are presented in Supplementary Table 1. RNA reverse-transcription and quantification were performed using the iTaq™ Universal SYBR^®^ Green One-Step Kit (Bio-Rad, Hercules, CA, United States) in a CFX96 Real-Time PCR Detection System (Bio-Rad) as follows: reverse transcription for 10 min at 50°C, polymerase activation, and DNA denaturation for 1 min at 95°C, and 40 cycles of denaturation at 95°C for 10 s followed by annealing/extension for 30 s at 60°C. Fluorescence was measured after each cycle. After cycling, melt curve analysis was performed from 65 to 95°C with 0.5°C increments every 2 s.

### Levels of NAD^+^, NADH, NADP^+^, and NADHP After Treatment With Sodium Hypochlorite or Chlorine Dioxide

After the 15-min treatments and neutralization with Na_2_S_2_O_3_, individual levels of NAD^+^, NADH, NADP^+^, and NADH were measured using the luciferase-based NAD/NADH-Glo™ and NADP/NADPH- Glo™ kits (Promega, Madison, WI, United States). Following manufacturer-provided protocols, 50 μl of cells in phosphate buffered saline (PBS) were lysed using 50 μl 0.2 N NaOH with 1% dodecyltrimethylammonium bromide (DTAB). For NAD^+^ or NADP^+^ analysis, 50 μl of lysed cells were combined with 25 μl of 0.4 N HCl and heated at 60°C for 15 min. After heating, the sample was incubated at room temperature for 10 min, and 25 μl of 0.5 M Trizma Base was added. For NADH or NADPH analysis, 50 μl of lysed cells were heated at 60°C for 15 min. After heating, the samples were incubated at room temperature for 10 min, and 50 μl of 0.2 N HCl/0.25 M Trizma Base was added. Fifty microliters of each sample were combined with 50 μl of NAD/NADH-Glo™ or NADP/NADPH-Glo™ detection reagent for NAD^+^/NADH or NADP^+^/NADPH, respectfully, in individual wells of 96-well white-walled tissue culture plate. The plate was placed in the dark for 30 min, and relative luminescence (RLU) was measured using a Synergy HTX plate reader (Biotek, Winooski, VT, United States).

### Data Analysis

The relative expression of individual genes was analyzed using the 2^–ΔΔCt^ method (Livak and Schmittgen, 2001). All experiments were performed in triplicate, and statistical analysis was performed using JMP (ver.12) or Sigmaplot (ver. 14) software with α = 0.05. Raw bacterial counts were log-transformed, and log reductions were determined by subtracting the populations of bacteria recovered after treatment from untreated controls. One-way ANOVAs coupled with Tukey’s HSD *post-hoc* tests were used to determine significant differences in viable cell counts, ROS levels, relative gene expression, and cofactor levels among the treatment conditions.

## Results

### Bacterial Viability and General Stress After Treatment

**FIGURE 1:**
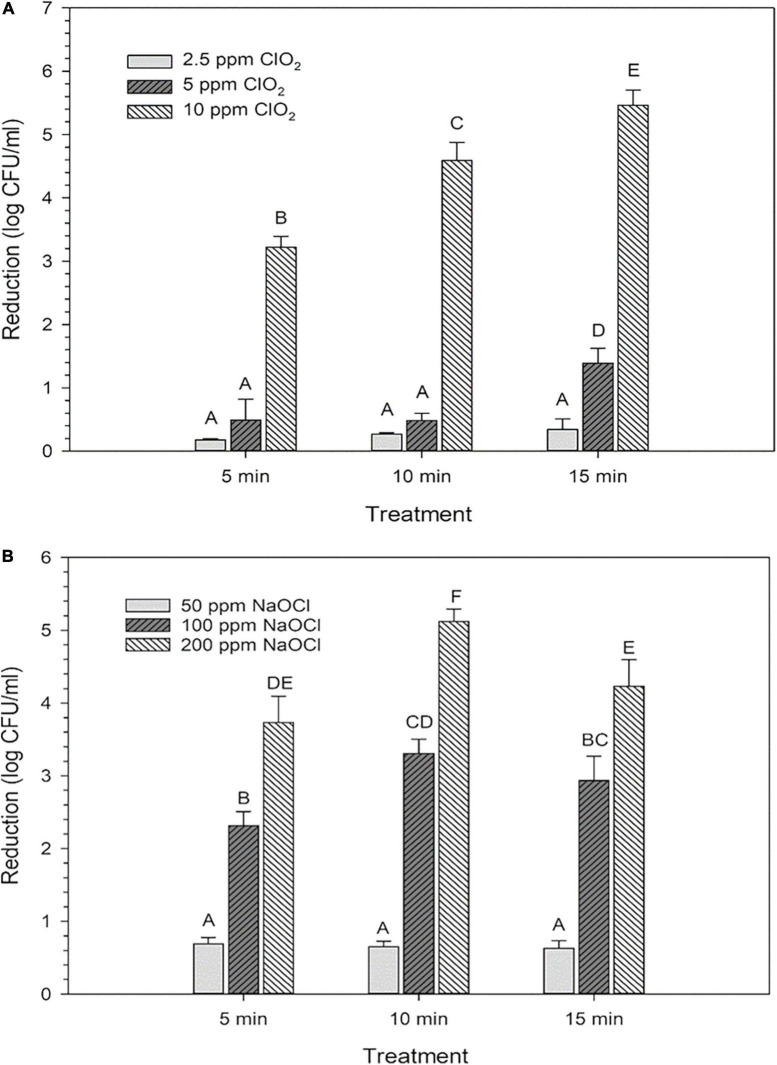
Log reductions of *Escherichia coli* O157:H7 after 2.5, 5, or 10 ppm ClO_2_ treatment for 5, 10, or 15 min compared **(A)** and treatment with 50, 100, or 200 ppm NaClO for 5, 10, or 15 min **(B)**. Data are presented as means ± standard deviations, and significant differences (*P* ≤ 0.05) in bacteria reductions observed after each treatment are represented by different letters (e.g., A–F).

The average reductions of *E. coli* after treatments with ClO_2_ or NaClO are presented in Figure 1. For ClO_2_, maximum log reductions of 0.3, 1.4, and 5.5 were achieved after 15 min treatments with 2.5, 5, or 10 ppm treatments, respectively (Figure 1A). All NaClO treatments achieved their maximum reductions at different time points, with 50 ppm reaching 0.69 after 5 min, while 100 and 200 ppm treatments resulted in maximum log reductions of 3.3 and 5.1, respectively, after 10 min (Figure 1B).

Monitoring gene expression to general stress as a control was performed by measuring levels of *uspA* mRNA. Expression of *uspA* increased accordingly with NaClO concentration, indicating a concentration-dependent stress response (Table 1). However, there was no change in *uspA* expression after ClO_2_ concentration increased. Levels of *rpoS* and *ompC* expression were additionally monitored to observe any potential changes in the regulation of stress responses or the presence of osmotic stress. Expression of *rpoS* decreased from 1.1 log to 0.3 log as NaClO concentration increased, while ClO_2_ treatment resulted in either base-level or downregulation of *rpoS*. The only examined gene that showed a similar trend after both treatments was *ompC*, indicating that both treatments resulted in osmotic stress.

**TABLE 1:** Log relative expression of select genes after treatment of *Escherichia coli* O157:H7 with NaClO or ClO_2_.

		Relative gene expression (log 2^–ΔΔCt^)					
		
Sanitizer	Concentration (ppm)	*sodA*	*oxyR*	*soxR*	*uspA*	*rpoS*	*ompC*
NaClO	50	0.2 ± 0.1^AD^	−0.1 ± 0.4^A^	−1.1 ± 0.2^A^	−0.3 ± 0.1^A^	1.1 ± 0.3^A^	−0.2 ± 0.4^A^
	100	1.2 ± 0.1^BC^	0.9 ± 0.1^BC^	1.1 ± 0.1^B^	0.3 ± 0.2^AB^	0.4 ± 0.1^B^	0.3 ± 0.2^AB^
	200	1.8 ± 0.4^B^	1.2 ± 0.4^B^	2.1 ± 0.5^C^	0.9 ± 0.1^B^	0.3 ± 0.1^B^	0.9 ± 0.1^B^
ClO_2_	2.5	0.8 ± 0.4^AC^	0.3 ± 0.2^AC^	1.3 ± 0.1^B^	0.0 ± 0.3^A^	−0.4 ± 0.1^CD^	0.1 ± 0.2^A^
	5	0.0 ± 0.4^D^	0.0 ± 0.2^A^	−0.1 ± 0.1^D^	−0.2 ± 0.3^A^	−0.9 ± 0.3^C^	−0.0 ± 0.2^A^
	10	0.0 ± 0.2^D^	−0.2 ± 0.4^A^	−0.1 ± 0.1^D^	0.2 ± 0.2^A^	0.0 ± 0.3^BD^	1.0 ± 0.3^B^

*Significant differences (P ≤ 0.05) in relative gene expression observed after each treatment are represented by different letters (e.g., ^A–D^).*

### Internal Reactive Oxygen Species Levels and Expression of Key Reactive Oxygen Species-Stress Genes After Treatment

Expression of select genes involved in ROS defense (*sodA*, *oxyR*, *soxR*) were all significantly (*P* > 0.05) upregulated after exposure to 100 and 200 ppm NaClO indicating that the cells were responding to the ROS stress at a gene expression level (Table 1). For ClO_2_ treatments, there were only 0.8, 0.3, and 1.3-fold increases for *sodA*, *oxyR*, and *soxR* expression, respectively, after treatment with 2.5 ppm. After treatment with 5 or 10 ppm ClO_2_, there were no significant changes in expression of the examined ROS-stress genes. Because there were significant log reductions after the 5 and 10 ppm treatment and little change in ROS-stress gene expression observed, these findings indicate that the cells are responding to NaClO and ClO_2_ treatments differently at the gene expression level.

**FIGURE 2:**
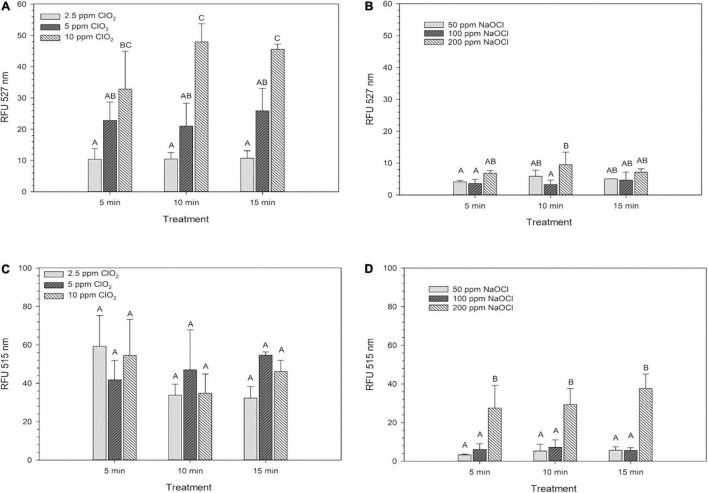
Intracellular ROS of *Escherichia coli* O157:H7 measured by DCF-DA after treatment with ClO_2_ **(A)** or NaClO **(B)** and the ROS levels as measured by APF after treatment with ClO_2_ **(C)** or NaClO **(D)**. Data are presented as means ± standard deviations, and significant differences (*P* ≤ 0.05) in relative fluorescence units (RFUs) observed after each treatment are represented by different letters (e.g., A–C).

Internal ROS measured by 2′,7′–dichlorofluorescin diacetate (DCFDA) after ClO_2_ significantly increased (*P* < 0.05) as the treatment concentration increased. However, increasing the exposure time did not influence internal ROS levels after ClO_2_ treatment (Figure 2A). Compared to ClO_2_ treatment, the ROS measured by DCFDA after NaClO treatment was relatively minimal (Figure 2B). The levels of ROS as measured by aminophenyl fluorescein (APF) did not significantly change with ClO_2_ treatment concentration (Figure 2C). In comparison, there was a significant increase in ROS levels measured by APF after the 200 ppm NaClO treatments (Figure 2D).

### Levels of NAD^+^ and NADH After Treatment

**FIGURE 3:**
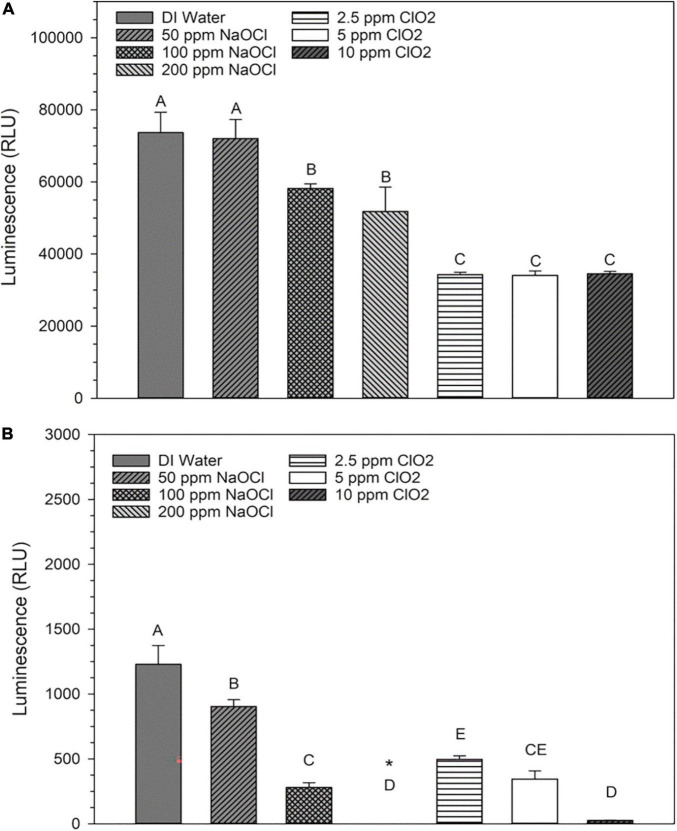
Relative levels of NAD^+^ **(A)** or NADH **(B)** present in *Escherichia coli* O157:H7 after treatment with NaClO or ClO_2_. Data are presented as means ± standard deviations, and significant differences (*P* ≤ 0.05) in relative luminosity units (RLUs) observed after each treatment are represented by different letters (e.g., A–E). The symbol “*” represents that the RLU was below the detection limit of the assay.

The changes in base levels of NAD^+^ are presented in Figure 3A. The 50 ppm NaClO treatment resulted in an insignificant (*P* > 0.05) decrease in luminosity compared to distilled water, while both the 100 and 200 ppm concentrations had significant (*P* < 0.05) decreases. All three ClO_2_ treatments resulted in similar NAD^+^ levels. Also, there were significant decreases in NADH luminosity intensity as concentration increased for both NaClO and ClO_2_ (Figure 3B). Despite these changes, the overall ratio of NAD^+^:NADH remained relatively the same, with 99% ≥of the cofactor in the oxidized form (NAD^+^) and ≤1% in the reduced (NADH) state for all ClO_2_ or NaClO treatments (Table 2).

**TABLE 2:** The average percentage of reduced or oxidized forms of NAD^+/^NADH or NADP^+^/NADPH were measured after treatment with ClO_2_ or NaClO.

Sanitizer	Concentration (ppm)	NAD^+^ (%)	NADH (%)	NADP^+^ (%)	NADPH (%)
DI Water	0	98	2	29	71
ClO_2_	2.5	99	1	51	49
	5	99	1	72	28
	10	>99	<1	86	14
NaClO	50	99	1	40	60
	100	>99	<1	55	45
	200	>99	<1	96	4

### Levels of NADP^+^ and NADPH After Treatment

**FIGURE 4:**
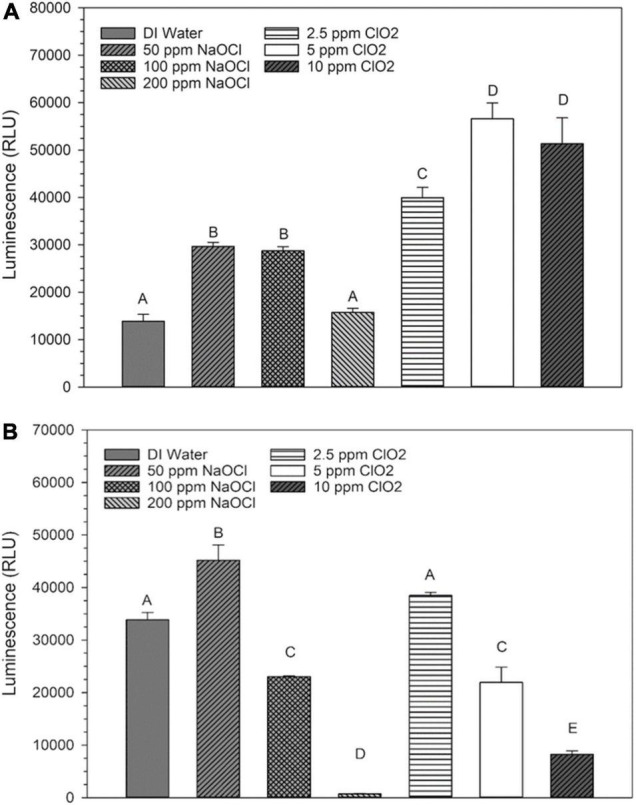
Relative levels of NADP^+^ **(A)** or NADPH **(B)** present in *Escherichia coli* O157:H7 after treatment with NaClO or ClO_2_. Data are presented as means ± standard deviations, and significant differences (*P* ≤ 0.05) in relative luminosity units (RLUs) observed after each treatment are represented by different letters (e.g., A–E).

Changes in NADP^+^ levels after NaClO treatment are shown in Figure 4A. Compared to the distilled water control treatment, there was an increase in NADP^+^ luminosity after treatment with 50 or 100 ppm NaClO. Conversely, the 200 ppm treatment resulted in NADP^+^ luminosity similar to that of distilled water. All three ClO_2_ treatments resulted in an increased level of NADP^+^ compared to the control. Like the trend observed in NADH levels after treatment, NADPH levels significantly decreased as concentration increased for both treatments (Figure 4B). Additionally, as both ClO_2_ and NaClO increased in concentration, there was a shift in the NADP^+^:NADPH ratio favoring the reduced state (Table 2). After 2.5 ppm ClO_2_ treatment, the NADP^+^:NADPH ratio was 51:49% which shifted to 86%:14% after the 10 ppm treatment. Similarly, there was a shift from 40:60% to 96:4% as the NaClO treatment concentration increased.

## Discussion

**FIGURE 5:**
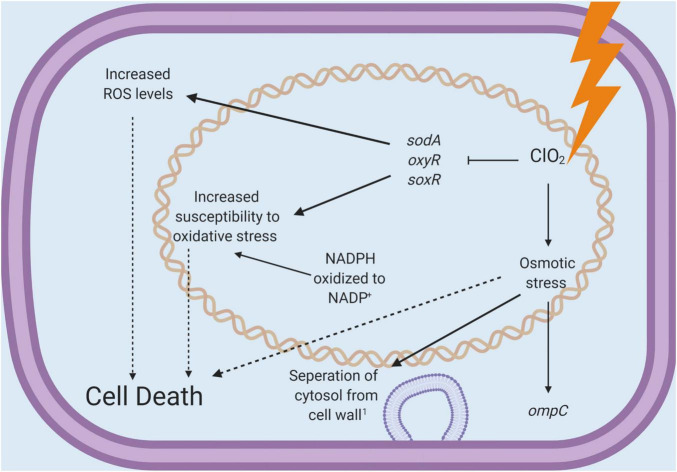
A schematic diagram for the antibacterial mechanisms of chlorine dioxide concluded from the present study.

The present study demonstrates the distinctions between two commonly used chlorinated water treatment sanitizers. Management of ROS is vital to the survival of every cell, and measuring relative levels of intracellular ROS can provide insight into the physiological state of a cell. In this study, ClO_2_ treatments resulted in increased concentrations of measurable ROS without significantly increasing ROS-related gene expression. In comparison, exposure to *E. coli* O157:H7 with NaClO elicited a clear transcriptional response to oxidative stress, presumably resulting in reduced intercellular ROS levels detected. It is possible that the increased expression of ROS-defense genes ultimately allowed the cells to reduce the internal levels of ROS after NaClO treatment. In contrast, with ClO_2_, there was relatively little ROS-defense gene activity and a noticeable increase in ROS levels. Based on these results alone, it appears that ClO_2_ and NaClO caused cell death utilizing different mechanisms (Figure 5). Furthermore, the observation that ClO_2_ treatment resulted in the downregulation of key ROS-defense genes and increased the levels of intracellular ROS directly implies that the antibacterial potency of ClO_2_ does not appear to be simply due to direct oxidation by ClO_2_. While ClO_2_ is an oxidizing agent, the antibacterial effect of ClO_2_ is more complicated than widespread oxidation, as observed with NaClO.

The membrane-permeable DCFDA is a general oxidative stress indicator that can provide cursory information on bacterial ROS responses. Upon entry to cells, it is cleaved by cellular esterases at ester bonds which produces a polar, membrane-impermeable product that, upon oxidation, becomes fluorescent. While DCFDA is most sensitive to H_2_O_2_, it can also react with other oxidants (Eruslanov and Kusmartsev, 2010). The other ROS probe used, APF, is non-fluorescent until it reacts with hydroxyl radicals (.OH), peroxynitrite (ONOO^–^), or hypochlorite (ClO^–^). The relative increase in probe signal observed after ClO_2_ treatment indicates an intracellular ROS response, but it is unknown whether this increase was due to the direct generation of ROS by the treatments or through interference with typical ROS-defense systems. The difference in results between the two probes could be due to the generation of different species of ROS during or after treatment, differences in membrane permeability of the probes, and limitations of the sensitivity of the probes in general. Previously, Cossu et al. (2017) found a non-linear relationship between DCFDA fluorescence and *E. coli* O157:H7 viability after treatment with H_2_O_2_ and no relationship between APF fluorescence and viability. Like the APF results in the present study, they also did not find significant increases in fluorescence corresponding to increases in sanitizer concentration; the difference in ROS levels after treatment with each sanitizer could imply that the increase in ROS levels after ClO_2_ treatment could be due to damage to ROS management and defense processes. These findings help contextualize how ClO_2_ treatment can result in higher levels of microbial inactivation in water which has major public health implications.

All aerobic microorganisms manage ROS that accumulates in cells as products of the incomplete reduction of molecular oxygen. In this study, the expression of manganese superoxide dismutase (*sodA*) was used as an indicator of oxidative stress in *E. coli* O157:H7. The expression of *sodA*, and the other two ROS-defense-related genes monitored in this study, *soxR*, and *oxyR*, can be influenced by other external environmental factors, which result in predictable cellular responses to oxidative stress. Expression of *sodA* itself is regulated by SoxR regulon. Upon activation by superoxide, SoxR activates the transcription of *soxS*, which, in turn, regulates critical genes responsible for managing oxidative stress, which includes *sodA*. After treatment with NaClO, a corresponding upregulation of s*odA* expression increased as treatment concentration increased, indicating that NaClO treatment caused dose-dependent superoxide stress in *E. coli* O157:H7. However, there was no significant increase in the expression of these genes after treatment with ClO_2_. Previously, Mei et al. (2017) observed that treatment of *E. coli* O157:H7 on lettuce with 50 mM H_2_O_2_ resulted in a decrease in the expression of *sodA*, *soxR*, and *oxyR*. Additionally, the expression of *soxR* was used to indicate the incidence of oxidative stress in the present study. After treatment with 2.5 ppm ClO_2_, there was a noticeable increase in *soxR* expression and a corresponding slight increase in *sodA* expression. This indicates the formation of superoxide because of treatment. However, as treatment concentration increased, the levels of expression of these two genes remained at baseline, which is counter to the observation of oxygen radical activity observed with DCFDA.

The third ROS-defense gene monitored in this study, *oxyR*, is transcribed after oxidation of the OxyR regulon. In the presence of H_2_O_2_, OxyR is oxidized and then activates transcription of the OxyR regulon genes, which function to protect the cell against H_2_O_2_ (Gray et al., 2013). Conversely, the expression of *oxyR* did not change after ClO_2_ exposure either, which could indicate that there was no successful activation of the OxyR regulon. The fact that there was a significant increase in intracellular ROS after ClO_2_ treatment, but little ROS-defense gene induction, potentially represents ClO_2_-induced disruption of the normal signaling pathways which could result in the upregulation of these genes. Therefore, cells treated with ClO_2_ could potentially be more susceptible to oxidative damage and ROS accumulation. These findings further support that ClO_2_ causes different cellular responses from what is observed with treatment with a typical oxidizer like NaClO.

To serve as controls for other stresses, the expression of three other stress-related genes (*uspA*, *rpoS*, and *ompC*) was measured as well. Like the results observed with the ROS-defense genes, expression of *uspA* increased with NaClO concentration while ClO_2_ treatment resulted in little change. While the exact functions of UspA proteins are unclear, increased expression of *uspA* has been observed after numerous types of stresses and has been associated with improved survival rates during stress in *E. coli* (Vollmer and Bark, 2018). Likewise, *rpoS* is associated with multiple types of stress resistances, and *rpoS* mutants have been previously described as being sensitive to treatment with oxidizers (Battesti et al., 2011). In the present study, the relative levels of *rpoS* expression inversely to NaClO concentration. Mei et al. (2017) previously also reported downregulation of *rpoS* after treatment of *Escherichia coli* O157:H7 with 50 mM H_2_O_2_. However, it has been demonstrated that OxyS, a product of OxyR activation, negatively regulates *rpoS* translation (Zhang, 1998), indicating that the downregulation of *rpoS* observed in the present was a result of increased *oxyR* expression. The only investigated gene that demonstrated similar trends in expression after treatment with either NaClO or ClO_2_ was *ompC, indicating* that *E. coli* O157:H7 was undergoing osmotic stress during both types of treatments. Previously, Bridges et al. (2020) found that *E. coli* O157:H7 cells treated with 10 ppm ClO_2_ had visible sections where the cytoplasm was separated from the cell wall, further supporting that during high concentration ClO_2_ treatment, and NaClO as well, osmotic stress could contribute to bacterial mortality.

NADPH is essential for the function of catalases, superoxide dismutases, and glutathione peroxidases (Singh et al., 2008). The redox potential of NADP^+^/NADPH is almost identical to NAD^+^/NADH, but the key difference between them is that NADP^+^/NADPH is primarily used in anabolic redox reactions while NAD^+^/NADH is used in oxidation reactions (Spaans et al., 2015). In this study, the cofactor measurements after treatment demonstrate a shift in the overall ratio of NADP^+^:NADPH as the concentration of both antimicrobials increased. The average percentages of NADP^+^:NADPH after 50 ppm and 200 ppm NaClO treatments were 49 and 14%, while for 2.5 and 10 ppm ClO_2_, they were 60 and 4%. One possibility is that NADPH was oxidized directly during NaClO or the ClO_2_ exposure due to the abundance of NADP^+^ increasing with treatment concentration. However, given the differences in gene expression mentioned earlier, it is impossible to determine if the increased NADP^+^ levels are due to similar metabolic processes after each treatment. Regardless, these results do indicate the importance of NADPH in managing oxidative stress and are in agreement with other research on the topic. Christodoulou et al. (2018) previously demonstrated the rapid increase of NADPH during oxidative stress in *E. coli* and further explained the importance of NADPH regeneration *via* the pentose phosphate pathway. Additionally, Bitew et al. (2020) demonstrated that mutant *Coxiella burnetii* with diminished functionality of SdrA, an NADP regenerating enzyme, were more sensitive to oxidative stress *in vitro*.

Increased susceptibility to oxidative stress provides opportunities for significant damage to the lipid bilayer and other regions prone to oxidative damage. Similar studies to the present one have indicated that lipid peroxidation plays a key role in disrupting the membrane fluidity of the bacterial cells wall of gram-positive microorganisms, such as *Enterococcus faecalis* (Ersoy et al., 2019) as well as the gram-negative *E. coli* (Bridges et al., 2020). The subsequent byproducts of lipid peroxidation, such as aldehydes, can penetrate the cytoplasm and target many proteins and nucleic acids within the cell. These byproducts may also uncouple electron transport across a cellular membrane and decrease cellular ATP. In addition, it is important to consider the sublethal mechanism of the injury, which would affect genomic expression. Although the TAL recovery method was implemented to increase bacterial recovery, it is impossible to rule out that there were viable but non-culturable (VNBC) cells present after treatment. The presence of VNBC could be contributing to the variability between different time points observed in the plate-count data. Furthermore, evidence of resistance to ClO_2_ treatment in *E. coli* O157:H7 has been previously demonstrated, indicating that injured and/or stressed cells can effectively respond to treatment (Shu et al., 2020). It is also important to note that VNBC cells would also influence the values of the data discussed below and be valuably contributing to these physiological metrics. Future studies will investigate the sublethal mechanism of bacterial recovery, especially in the presence of important antioxidants such as glutathione.

Sodium hypochlorite is an inexpensive sanitizer currently used in mass quantities for agriculture and municipal water treatment. Comparatively, the cost of generation, storage, and monitoring of alternative sanitizers, such as ClO_2_, are currently more expensive. However, the cost per liter or square meter is not the only area growers need to consider when applying pathogen mitigation strategies. There are several drawbacks to the ubiquitous use of NaClO, such as reduced antimicrobial efficacy and the formation of toxic byproducts. Therefore, the USDA National Organic Standards has formally recommended that the application dosage for hypochlorites to agricultural water not exceed the 4 ppm residue limit imposed by the Safe Drinking Water Act (Tiemann, 2014). In addition, NaClO has limited efficacy outside of specific pH and organic load. Furthermore, the reduced antimicrobial efficacy can lead to subpopulations of pathogenic microorganisms persisting in the environment and antibiotic resistance. Therefore, alternative sanitizers such as ClO_2_ can be a tool to consider for agricultural water safety because of the antimicrobial range at a significantly lower residual level.

## Data Availability Statement

The original contributions presented in this study are included in the article/Supplementary Material, further inquiries can be directed to the corresponding author.

## Author Contributions

DB performed experiments and wrote the draft of the manuscript. AL edited the drafts of the manuscript. VW conceived and supervised the study, designed experiments, and reviewed and edited drafts of the manuscript. All authors read and approved the final draft of the manuscript.

## Conflict of Interest

The authors declare that the research was conducted in the absence of any commercial or financial relationships that could be construed as a potential conflict of interest.

## Publisher’s Note

All claims expressed in this article are solely those of the authors and do not necessarily represent those of their affiliated organizations, or those of the publisher, the editors and the reviewers. Any product that may be evaluated in this article, or claim that may be made by its manufacturer, is not guaranteed or endorsed by the publisher.
