# Supplementary material for: Fundamental Differences in Inactivation Mechanisms of Escherichia coli O157:H7 Between Chlorine Dioxide and Sodium Hypochlorite
Source: Front Microbiol. 2022 Jun 17;13:923964. doi: 10.3389/fmicb.2022.923964 (PMC9247566; doi:10.3389/fmicb.2022.923964)
Supplement: Supplementary file 1 [file Data_Sheet_1.PDF]

Table 1S. Primers used for real-time PCR reactions

Target	Sequence (5’-3’)	Reference
16S rRNA	F: AGAGGATGACCAGCCACAC	Mei et al. 2015
	R: CGGGTAACGTCAATGAGCAAAG	
sodA	F: ACCACACCAAACACCATCAGAC	Mei et al. 2015
	R: GTGACCGCCAGCGTTGTTG	
oxyR	F: GAAGCACAGACCCACCAGTT	Mei et al. 2015
	R: CAAACAACGGCACTTCAATG	
soxR	F: CGTAACAGCGGCAATCAGC	Mei et al. 2015
	R: ACGCCAAACGCTTCACCAAT	
uspA	F: AGGCTACCCAATCACTGAAACC	Mei et al. 2015
	R: GCAGCGGAACAATCAGCATATC	
rpoS	F: AATCGTGGTCTGGCGTTGC	Mei et al. 2015
	R: GCGTATGTTGAGAAGCGGAAAC	
ompC	F: GAAACTGCAGCACCGAT	Yang et al. 2018
	R: CTTTGCTGTTCAGTACCAGG	
